# Epidemiological and Entomological Study After the Possible Re-Emergence of Dengue Fever in Croatia, 2024

**DOI:** 10.3390/microorganisms13030565

**Published:** 2025-03-02

**Authors:** Alan Medić, Vladimir Savić, Ana Klobučar, Maja Bogdanić, Marcela Curman Posavec, Diana Nonković, Ljubo Barbić, Ivana Rončević, Vladimir Stevanović, Tatjana Vilibić-Čavlek

**Affiliations:** 1 Department of Epidemiology, Zadar County Institute of Public Health, 23000 Zadar, Croatia; 2 Department of Health Studies, University of Zadar, 23000 Zadar, Croatia; 3 Poultry Center, Croatian Veterinary Institute, 10000 Zagreb, Croatia; v_savic@veinst.hr (V.S.); roncevic@veinst.hr (I.R.); 4 Department of Epidemiology, Andrija Štampar Teaching Institute of Public Health, 10000 Zagreb, Croatia; ana.klobucar@stampar.hr (A.K.); marcela.curman@stampar.hr (M.C.P.); 5 Department of Virology, Croatian Institute of Public Health, 10000 Zagreb, Croatia; maja.bogdanic@hzjz.hr; 6 School of Medicine, University of Zagreb, 10000 Zagreb, Croatia; 7 Department of Epidemiology, Split-Dalmatia County Institute of Public Health, 21000 Split, Croatia; diana.nonkovic@nzjz-split.hr; 8 Department of Health Studies, University of Split, 21000 Split, Croatia; 9 Department of Microbiology and Infectious Diseases with Clinic, Faculty of Veterinary Medicine, University of Zagreb, 10000 Zagreb, Croatia; ljubo.barbic@vef.hr (L.B.); vladostevanovic@gmail.com (V.S.)

**Keywords:** dengue, humans, mosquitoes, Croatia

## Abstract

Autochthonous dengue cases have been continuously recorded in Europe in the past two decades. The first autochthonous dengue case in Croatia was reported in 2010 on the Pelješac Peninsula, while imported cases were recorded continuously thereafter. In 2024, dengue re-emerged in Croatia. An epidemiological and entomological study was conducted after receiving information on dengue virus (DENV) infection in a German tourist probably acquired on Dugi Otok Island in Croatia in May 2024. Serum samples were collected from 30 residents of the Veli Rat region where the patient had stayed. In addition, mosquitoes were collected in the same area. Human samples were tested for the presence of DENV antibodies (ELISA and IFA) and DENV RNA (RT-qPCR), while mosquito samples were tested for DENV RNA (RT-qPCR). DENV IgM or IgG antibodies were found in 8 serum samples, while no one sample was RT-qPCR positive. No cross-reactivity with flaviviruses was detected in seropositive samples, supporting DENV infection. One patient was classified as a confirmed dengue case (IgG seroconversion in paired serum samples) and five as probable cases (IgM detection in a single serum sample). One additional patient, sampled only once, was IgG seropositive. Two of the seropositive individuals reported fever and rash three weeks before testing. The re-emergence of dengue in Croatia highlights the need for continuous monitoring of DENV circulation in both humans and vectors.

## 1. Introduction

Dengue virus (*Orthoflavivirus dengue*; DENV) is an emerging and one of the most widespread arboviruses that belongs to the family *Flaviviridae*, genus *Orthoflavivirus*. Four main DENV serotypes (DENV 1–4) circulate in tropical and subtropical regions of Africa, Asia, the Pacific, the Americas, and the Caribbean, frequently in urban or semi-urban areas [1]. In nature, DENV is maintained in an enzootic (sylvatic) cycle involving non-human primates and mosquito vectors. The rural endemic cycle occurs in small villages where the virus transmission is contained. In the urban cycle, the virus is transmitted between humans by the bites of *Aedes aegypti* and *Aedes albopictus* mosquitoes causing periodic epidemics [2,3].

The invasive mosquito species *Ae. albopictus* is a major public health concern in Europe because of its vector capacity for emerging arboviruses, including DENV [4]. In Croatia, *Ae. albopictus* was first discovered in 2004 in the southwest suburbs of Zagreb [5]. In the following years, it was the most prevalent species in coastal regions from Istria in the north to Dalmatia and the islands to Dubrovnik in the south [6,7]. This species was also detected and established in northwestern and eastern continental regions [8,9,10].

Around 80% of primary human DENV infections are asymptomatic, while less than 20% of infected individuals present as dengue fever or dengue hemorrhagic fever (DHF). The incubation period of dengue varies from 3 to 10 days, with an average of 4–7 days. Dengue fever is an acute febrile disease characterized by headache, retro-orbital pain, myalgia, arthralgia, and rash. DHF is a more severe disease characterized by fever and hemorrhagic manifestations (petechiae, ecchymoses, or purpura, bleeding from the gastrointestinal tract and other locations) [11]. Heterotypic secondary DENV infection may progress to dengue shock syndrome (DSS), leading to increased vascular permeability and multiorgan failure with a high case fatality rate [12].

As dengue is not endemic in the mainland European Union/European Economic Area (EU/EEA)**,** travelers infected outside the EU/EEA account for most cases. From 2014 to 2022, between 428 and 4363 imported cases were reported annually in returning travelers [13]. However, since 2010, autochthonous DENV cases have been continuously recorded, except for 2011, 2012, 2016, and 2017 [14]. More than 14 million dengue cases and 10,000 dengue-related fatalities have been reported worldwide since the beginning of 2024. Italy (213 cases), France (83 cases), and Spain (8 cases) have recorded autochthonous cases in mainland Europe [15].

In Croatia, sporadic imported dengue infections have been continuously recorded in travelers returning from endemic areas. In 2010, the first autochthonous dengue infection was recorded in a German tourist who stayed on the Pelješac Peninsula, followed by two additional cases in residents of the same village where the German patient had visited. Subsequent serological investigation of 14 healthy neighbors of the autochthonous case showed DENV IgG antibodies in three individuals, while six individuals were positive for both IgM and IgG antibodies. A further 112 samples tested from anonymous patients who visited a medical center for various reasons during October 2010 showed DENV IgG and/or IgM antibodies in 6 samples [16]. During 2011 and 2012, a seroepidemiological study was performed on asymptomatic individuals from seven counties in the Croatian littoral and four continental counties. The seroprevalence rates were found to be 0.55% among inhabitants of coastal and 0.72% in northeastern continental regions. The seropositivity was highest (2.2%) in Dubrovnik-Neretva County, where autochthonous dengue cases were reported in 2010. In addition, 3699 mosquitoes were collected at the Croatian littoral, but no evidence of DENV RNA was detected [17]. No indigenous DENV transmission was observed in Croatia thereafter.

This study reports the autochthonous dengue fever in a German tourist who stayed on Dugi Otok Island in Croatia in May 2024. DENV infection was further supported by serologic evidence of recent DENV infection in seven residents of the same geographic area. Indigenous dengue transmission in Croatia highlights the need for continuous monitoring of the possible virus circulation in non-endemic areas where competent mosquito vectors are established.

## 2. Materials and Methods

### 2.1. Dengue Case Description and Active Case Finding

**Figure 1:**
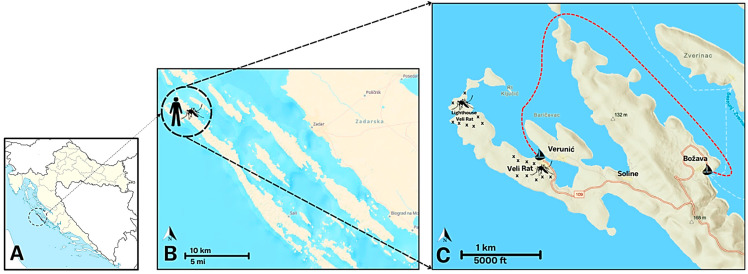
Sampling area on Dugi Otok Island. (**A**) Geographic location of Dugi Otok Island; (**B**) Human and mosquito sampling area in the Veli Rat; (**C**) Dengue-positive German tourist boat movement (presented with a red dashed line). X represents mosquito sampling micro-localities.

On 10 June 2024, the Epidemiological Department of the Zadar County Institute of Public Health was notified by the Munich Health Department of a German citizen, who developed symptoms of dengue fever immediately after returning to Germany from a 13-day stay on the Dugi Otok Island in Croatia. Geographically, the island is located west of Zadar, off the Dalmatian coast. At 114 square kilometers, it is the largest and westernmost of the Zadarian islands (Figure 1A). According to the information received from the patient, he came from Munich, Germany to Dugi Otok by car on 18 May 2024. The patient arrived at Veli Rat (one of 12 villages on the island) on 19 May and stayed on the island for 13 days. After setting out with the ship on 24 May, he sailed and stayed in the northwestern part of the island (Figure 1C) for five days. He returned to the Veli Rat on May 29. On May 30, he returned to Germany, where he developed the first symptoms that evening. The patient had a high fever (>38 °C) and a rash, and the serology testing conducted in Germany indicated dengue infection (positive DENV IgM antibodies). The patient reported no recent travel outside Croatia in the period of two weeks before symptom onset. In addition, he stated that he noticed a large number of mosquitoes in the Veli Rat marina’s bathrooms.

As this was the dengue case probably acquired in Croatia (the patient stayed in Croatia during the incubation period which is typically up to 10 days), the epidemiological investigation began in mid-June. In the following weeks, blood samples were collected from residents living in the Veli Rat region throughout the year. Sampling was offered to all residents (*n* = 100), but 30 (30%) individuals agreed to participate and were included in the study (convenience sample). Human sampling was conducted in four villages (Veli Rat, Verunić, Soline, and Božava; Figure 1C) on 15 September. Following the explanation of the aims of the DENV screening, all participants who consented to participate in the study filled out a questionnaire about the epidemiological history and the presence of symptoms related to dengue. In addition, mosquitoes were collected for entomological investigation (Figure 1B).

### 2.2. Mosquito Sampling

Mosquito samplings were conducted in a place of probable DENV transmission, on 31 July and 18 September in Veli Rat at two locations, in the marina, and around the lighthouse (Figure 1C, Table 1). Since the German tourist spent most of his vacation in the Veli Rat area and only briefly visited Verunić (very close to Veli Rat marina) and Božava while sailing by boat, Veli Rat marina and lighthouse were selected for sampling. At each location, there were several mosquito-sampling micro-localities. A total of 28 sampling occasions were gathered.

Mosquitoes were collected using BG-Sentinel traps (Biogents AG, Regensburg, Germany) with BG-Lure attractant. The traps were placed in the area of patients’ residence and movement. Sampling was performed during the afternoon, from 3:00 p.m. to 8:00 p.m.

The mosquitoes were transported to the laboratory in containers with dry ice, transferred to plastic tubes, and stored on dry ice until identification. Mosquitoes were morphologically identified by species or species complex on a chilling surface under a stereomicroscope, using the determination key by Becker et al. (2020) [18]. Female *Ae. albopictus* specimens collected at each micro-location were pooled and stored at −80 °C until virological testing.

### 2.3. Virology Testing

The Reference Center for Diagnosis and Surveillance of Viral Zoonoses of the Croatian Ministry of Health and the National Reference Laboratory for Arboviruses at the Department of Virology, Croatian Institute of Public Health was responsible for laboratory testing of human samples. Mosquito testing was performed at the Croatian Veterinary Institute.

DENV-specific IgM and IgG antibodies in serum samples were detected using a commercial enzyme-linked immunosorbent assay (Anti-Dengue virus ELISA IgM/IgG; Euroimmun, Lübeck, Germany) and interpreted as follows: IgM (ratio) < 0.8 negative, 0.8–1.1 borderline, >1.1 positive; IgG (RU/mL) <16 negative, 16–22 borderline, >22 positive. To exclude cross-reactivity with other flaviviruses, initially reactive samples were retested using a commercial ELISA for the detection of Usutu virus (USUV; Anti-Usutu virus ELISA IgG; Euroimmun, Lübeck, Germany) and a commercial indirect immunofluorescence assay (IFA; Flavivirus Mosaic; Euroimmun, Lübeck, Germany) for the detection of tick-borne encephalitis virus (TBEV), West Nile virus (WNV), Japanese encephalitis virus (JEV), and yellow fever virus (YFV). Specific fluorescence at a dilution of 1:10 for IgM and 1:10 (TBEV, WNV, JEV) or 1:100 (YFV) for IgG was considered a positive result.

DENV RNA was detected using a real-time reverse transcription-polymerase chain reaction (RT-qPCR) for the detection of all DENV serotypes [19] with changes in usage of the RNA extraction kit and the RT-qPCR kit. Briefly, a High Pure Viral Nucleic Acid Kit (Roche Applied Science, Penzberg, Germany) was used for the RNA extraction and the Brilliant III Ultra-Fast QPCR Master Mix (Agilent Technologies, Santa Clara, CA, USA) was used for the RT-qPCR according to the manufacturer’s protocols. The following primers and probe were used: forward primer (DF): 5′-AGG ACY AGA GGT TAG AGG AGA-3′, reverse primer (DR): 5′-CGY TCT GTG CCT GGA WTG AT-3′ and probe (DP): FAM-5′-ACA GCA TAT TGA CGC TGG GAR AGA CC-3′-TAMRA.

Patients were classified according to the European Center for Disease Control and Prevention; ECDC EU case definitions epidemiological (residence in an area with documented ongoing dengue transmission, within the two weeks before symptom onset) and laboratory criteria as (a) probable case (detection of DENV-specific IgM antibodies in a single serum sample); (b) confirmed case (isolation of DENV, detection of DENV RNA, detection of DENV antigen, detection of DENV IgM antibodies in a single serum sample confirmed by virus neutralization test (VNT) or seroconversion/four-fold increase in DENV antibodies in paired serum samples) [20].

### 2.4. Statistical Analysis

Study participants are described by age, gender, epidemiological history, and clinical presentation using descriptive statistics. The age of the participants is expressed as median and range. Categorical variables are expressed as frequencies and percentages.

## 3. Results

### 3.1. Human Testing

Demographic and clinical data of participants included in the study are presented in Table 2. All participants recorded frequent mosquito bites. Nine out of thirty (30%) tested individuals reported clinical symptoms within 4 weeks before sampling. Six participants reported fever, one reported a rash, and two reported fever and rash. None of the participants reported a history of traveling abroad in the last six months, while one was vaccinated against yellow fever.

Eight participants tested positive for DENV IgM or IgG antibodies using ELISA. Serology and RT-PCR results of eight DENV seropositive participants are presented in Table 3. According to the ECDC EU Case definitions (dengue laboratory criteria) [20], one patient (case 3) was classified as a confirmed case (seroconversion in paired serum samples) and five (cases 1, 2, 4–7) as probable cases (IgM detection in a single serum sample). In addition, one patient was IgG seropositive (case 8), while one (the patient vaccinated against YF) had borderline DENV IgG result in paired serum samples (case 9). DENV RNA was not detected in any sample. Two of the seropositive individuals (cases 2 and 7) reported fever and rash three weeks before testing.

Eight tested samples from dengue seropositive individuals (Table 3) were negative for USUV using ELISA and TBEV, WNV, JEV, and YFV IgM and IgG antibodies using IFA (cases 1–8). In a sample with a borderline dengue IgG result (case 9), USUV, TBEV, WNV, and JEV antibodies were negative, while IgG titer to YFV of 100 was found, indicating a postvaccinal immune response (Supplementary Table S1).

**Figure 2:**
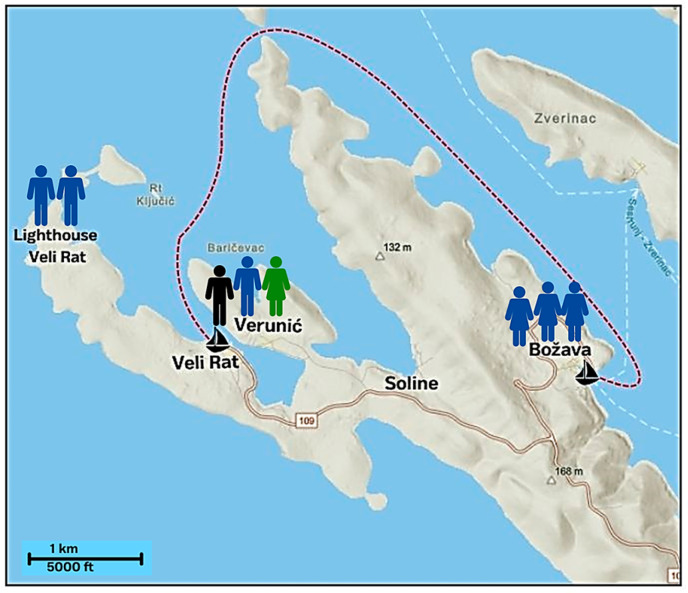
Geographic distribution of dengue seropositive individuals in the villages of Veli Rat surroundings: black silhouette–confirmed case (seroconversion in paired serum samples), blue silhouettes–probable cases (IgM detection in a single serum sample), green silhouette–IgG seropositive (in a single serum sample). A red dashed line represents dengue-positive German tourist boat movement.

The geographic distribution of DENV seropositive residents of Dugi Otok Island is presented in Figure 2. Seropositive individuals were from three villages in the northwestern part of the island (Veli Rat, Soline, and Božava).

### 3.2. Mosquito Testing

A total of 186 mosquitoes were collected at two locations (17 micro-locations) in Veli Rat. Two mosquito species were identified, *Ae. albopictus* (63 specimens) and *Culex pipiens* complex (123 specimens). Female *Ae. albopictus* specimens (55) were pooled in 16 pools (Table 4) and tested for the presence of DENV RNA using an RT-qPCR. No one DENV-positive pool was detected.

### 3.3. Public Health and Vector Control Measures

After receiving information about the autochthonous dengue fever in the area of Veli Rat, an authorized public health enforcement company was contacted to perform an initial mosquito control treatment in the suspected area. Larvicide treatment with silicone-based liquid (physical mechanism of action) and the adulticide treatment by using cypermethrin-tetramethrin in cold ultra-low-volume application were performed. In addition, breeding sites were removed if possible. The measures were taken in the whole urban area of Veli Rat, especially in the nautical marina and the lighthouse area, which is a tourist attraction. On several occasions, the local population was informed about dengue, its transmission, and symptoms.

## 4. Discussion

Although most dengue cases in Europe are imported, a significant increase in locally transmitted infections has been observed, which occur when competent mosquito vectors are established in European countries. In 2024, local dengue transmission was noted in three European countries (Italy, France, and Spain) [21].

Sporadic imported dengue infections were continuously recorded in Croatia; however, autochthonous infections were reported only in 2010 at the Croatian littoral (Pelješac Peninsula, South Dalmatia) [16]. According to the data of the Croatian Institute of Public Health, in 2024, imported dengue infection was reported in five Croatian residents who visited endemic areas.

The detection of dengue in a German tourist in 2024, probably acquired in Croatia, represents the first indigenous DENV transmission in Zadar County and Northern Dalmatia. Since DENV was not confirmed by molecular testing of humans and mosquitoes and no confirmation of seropositive residents of Dugi Otok by a VNT was performed, there was a possibility of false positive serology results. Nonetheless, no serological cross-reactivity with other flaviviruses (TBEV, WNV, USUV, JEV, YFV) was reported which further supported the evidence of DENV infection.

From August to October 2024, a large dengue outbreak with 199 cases occurred in the Marche Region in central Italy [22]. Considering that this region is only 100 km away by sea from Dugi Otok and that Italian tourists often visit the western part of Dugi Otok by boat, it is possible that DENV-infected viremic tourists from Italy introduced the virus in Croatia. Furthermore, it is possible that the virus was spread by boats carrying infected mosquitoes.

The re-emergence of dengue in Croatia is not unexpected due to the presence of *Ae. albopictus* and favorable climate conditions. The mean air temperature in Zadar County in May and June 2024 was above the multiannual average (1991–2020), with an anomaly of 1.4 °C. According to percentile ranks and classification ratings, thermal conditions for May and June fall under the warm category (84.1 and 87.8 percentile, respectively). Analyzing the total monthly precipitation in May and June, the cumulative precipitation amount (mm) was lower (33%) than the multiannual average in May and higher (141%) in June, classified as dry in May (14.5 percentile) and rainy in June (75.7 percentile) [23].

Many studies have shown that temperature positively correlates with the number of dengue cases [24]. Longer seasons of higher temperatures provide more favorable conditions for mosquito survival and reproduction [25]. Temperature is closely related to the survival of the *Aedes* mosquito, the DENV replication, and the vector competence of mosquitoes to transmit the virus [24]. Temperatures between 20 °C and 26 °C are optimal for DENV transmission; however, transmission may occur within the 12–30 °C temperature range [26]. In general, the activity of *Ae. albopictus* increases with increasing temperature. The life cycle of *Ae. albopictus* shortens, and the population growth rate increases at higher temperatures. In addition, the extrinsic incubation period shortens at higher temperatures, suggesting a proportional relationship between temperature and dengue risk [27].

The seasonal activity of *Ae. albopictus* in Europe can explain the early detection of dengue in Croatia in 2024, at the end of May. Studies have shown that in southern Europe, *Ae. albopictus* can remain active for longer periods than in northern Europe. Around the Mediterranean Basin, it is expected that spring hatching will occur in late February, compared to early to mid-April in most of Western Europe. In Croatia, in the city of Zagreb with a moderate continental climate, oviposition activity begins in April (weeks 17–18) and completes in November (weeks 45–46). The highest number of eggs was detected from the beginning of July to mid-September [10]. In the Mediterranean Basin, the egg density peaks from the end of July to the end of September. The longest mosquito activity can be found, lasting from 39 to 43 weeks, which implies that the season in this area can potentially start at the end of February and last until the middle of November [28]. In a study conducted from 2009 and 2010 in Split area, Adriatic Croatia, oviposition activity started in April (week 13) and completed at the beginning of December (week 48) [7].

Dengue case peaks in Europe vary from year to year. The number of cases is highest in August and November, with occasional cases occurring in January and March–April as well. Both the seasonality of inbound travel and the seasonal transmission patterns in the probable countries of infection which are linked to favorable meteorological conditions are reflected in the observed peaks [29].

Most autochthonous dengue cases in Europe are found in suburban or urban, most touristic regions along the Mediterranean coast, but in France and Spain, large cities were also hotspots. It can be assumed that there are a significant number of unreported cases because dengue is frequently asymptomatic or a mild non-specific febrile disease that in many instances remains undiagnosed [30].

In Croatia, a small number of mosquitoes were collected in the Veli Rat area, all of which tested negative for DENV RNA. In both sampling months (July and September), the air temperature was high in Zadar, classified as extremely warm thermal conditions (98.2 percentile) in July and warm (79.8 percentile) in September. Extremely warm conditions (98.5 percentile) were observed in August 2024 as well [23]. The environment where the mosquitoes were captured is well maintained, without stagnant water pools. Furthermore, the summer months were very dry (in contrast to April and May), creating unfavorable conditions for mosquito development.

Vector reduction and population control are components of a comprehensive strategy needed for effective DENV management. Furthermore, reducing mosquito habitats is an essential component in the control of dengue. This approach focuses on eliminating or reducing mosquito breeding sites around houses. It is important to remove any standing water in areas like plant pots, buckets, tires, cans, pet water bowls, and clogged gutters [31]. During the implementation of mosquito control in Veli Rat, no such artificial breeding sites were found.

In addition, individual protection is crucial to reduce the risk of contracting dengue. To prevent mosquito bites, travelers should wear long-sleeved shirts and trousers, use repellents, and ensure that windows and doors are fitted with screens. Travelers who return from holidays with symptoms compatible with dengue such as fever, rash, myalgia, and arthralgia should seek medical attention promptly and notify the healthcare practitioner about their travel history [25].

Since no specific antiviral drugs are available for dengue, treatment includes symptomatic and supportive therapy. In endemic areas, vaccination would probably be an effective method of dengue management. Two licensed vaccines (Dengvaxia and Qdenga) are available for DENV, while several types of vaccines, including live-attenuated vaccines, inactivated, DNA vaccines, recombinant subunit, peptide-based, and mRNA vaccines are in clinical trials [32].

Real-time robust dengue surveillance is important to address concerns regarding possible undiagnosed cases, unrecorded travel movements, and misdiagnosis as other arboviruses to better control DENV transmission. These factors might lead to unrecognized disease transmission and establish a possible threat for local outbreaks in regions where the disease is not endemic [25].

There are some limitations of the study that need to be addressed. A small sample size and convenience sampling could, at least in part, impact the representativeness of the results. Therefore, further seroepidemiological studies on a larger number of participants are needed to improve statistical significance. In addition, the conduction of follow-up mosquito sampling could possibly verify continued virus circulation.

## 5. Conclusions

The climate suitability for the DENV transmission in Europe is already increasing and predicted higher temperatures in the future will favor conditions for the spread of DENV. In many parts of Europe, the climatic suitability index for the *Ae. albopictus* mosquitoes and the suitable season length are expected to increase [29]. Indigenous dengue transmissions in non-endemic countries such as Croatia highlights the need for continuous surveillance of humans and mosquitoes (the “One Health” perspective). Furthermore, public awareness promotion and health education about DENV transmission are important for dengue prevention and control. For this reason, it is necessary to carry out educational campaigns to sensitize the local population about this public health threat, as well as to introduce a more robust surveillance system to reduce the risk for residents and tourists through targeted mosquito management.

## Figures and Tables

**Table 1:** Mosquito sampling sites and species detected in Veli Rat, Dugi Otok Island.

Sampling Date	Location	Micro-Location	Mosquito Species	N Females	N Males
31 July 2024	Veli Rat, Marina	a	*Aedes albopictus*	3	0
		b	*Aedes albopictus*	4	0
		c	*Aedes albopictus*/*Culex pipiens* complex	4/1	0/0
		d	*Culex pipiens* complex	2	0
	Veli Rat, Lighthouse	a	*Aedes albopictus/Culex pipiens* complex	4/27	0/5
		b	*Aedes albopictus/Culex pipiens* complex	5/2	1/4
		c	*Aedes albopictus/Culex pipiens* complex	4/3	0/6
		d	*Aedes albopictus/Culex pipiens* complex	1/1	0/0
18 September 2024	Veli Rat, Marina	a	*Aedes albopictus/Culex pipiens* complex	1/1	0/0
		b	*Aedes albopictus/Culex pipiens* complex	3/5	0/0
		c	*Aedes albopictus/Culex pipiens* complex	1/2	0/0
		d	*Aedes albopictus*	8	0
		e	*Aedes albopictus*	1	0
	Veli Rat, Lighthouse	a	*Aedes albopictus/Culex pipiens* complex	1/1	0/1
		b	*Aedes albopictus/Culex pipiens* complex	6/6	6/0
		c	*Aedes albopictus*	3	1
		d	*Aedes albopictus/Culex pipiens* complex	6/56	0/0
		Total	*Aedes albopictus/Culex pipiens* complex	55/107	8/16

**Table 2:** Demographics, epidemiological, and clinical data of tested individuals.

Characteristic		N Tested = 30
Sex	Male	17 (56.66%)
	Female	13 (43.34%)
Epidemiological history	Age (years), median (range)	58.7 (33–83)
	Vaccination (yellow fever)	1 (3.33%)
	Mosquito bites	30 (100%)
	Traveling history	0 (0.00%)
Clinical symptoms	Fever	6 (20.00%)
	Rash	1 (3.33%)
	Fever + Rash	2 (6.66%)

**Table 3:** Serology and RT-PCR results of eight dengue seropositive individuals.

Case	Sex	Age	Clinical Symptoms	Sample	DENV IgM (Ratio)	DENV IgG (RU/mL)	DENV-RNA
1	F	55	Not reported	I	Positive (3.16)	Negative (5.72)	Negative
2	M	53	Fever	I	Positive (2.99)	Negative (<2)	Negative
3	M	81	Not reported	I II	Negative (0.31) Negative (0.23)	Negative (12.71) Positive (30.38)	Negative
4	M	56	Not reported	I	Positive (1.32)	Negative (5.87)	Negative
5	F	55	Not reported	I	Positive (1.62)	Negative (3.85)	Negative
6	F	63	Not reported	I	Positive (1.79)	Negative (<2)	Negative
7	M	61	Rash	I	Positive (1.97)	Negative (<2)	Negative
8	F	76	Not reported	I	Negative (0.35)	Positive (34.47)	Negative
9 *	M	52	Not reported	I II	Negative (0.26) Negative (0.21)	Borderline (17.33) Borderline (19.26)	Negative

DENV = dengue virus; RU = relative units; * YFV vaccination; I = first serum sample; II = paired serum sample (collected after three weeks).

**Table 4:** The number of *Aedes albopictus* female mosquitoes sampled and tested pools for dengue virus RNA.

Sampling Date	Locality	*Aedes albopictus* Specimens (N Female)	*Aedes albopictus* (N Pools)
31 July 2024	Veli Rat, marina	11	3
	Veli Rat, lighthouse	14	4
18 September 2024	Veli Rat, marina	14	5
	Veli Rat, lighthouse	16	4
	Total	55	16

## Data Availability

The original contributions presented in the study are included in the article; further inquiries can be directed to the corresponding authors.

## Supplementary Materials

The following supporting information can be downloaded at: https://www.mdpi.com/article/10.3390/microorganisms13030565/s1. Table S1. Results of flavivirus serology testing for cross-reactivity.

## Abbreviations

The following abbreviations are used in this manuscript:

DENV	Dengue virus
DHF	Dengue hemorrhagic fever
DSS	Dengue shock syndrome
EU/EEA	European Union/European Economic Area
RT-qPCR	Real-time reverse-transcription polymerase chain reaction
ECDC	European Center for Disease Control and Prevention
TBEV	Tick-borne encephalitis virus
WNV	West Nile virus
JEV	Japanese encephalitis virus
YFV	Yellow fever virus

## References

[1] Islam M.T., Quispe C., Herrera-Bravo J.M., Sarkar C., Sharma R., Garg N., Fredes L.I., Martorell M., Alshehri M.M., Sharifi-Rad J. (2021). Production, Transmission, Pathogenesis, and Control of Dengue Virus: A Literature-Based Undivided Perspective. Biomed. Res. Int..

[2] Frasca F., Sorrentino L., Fracella M., D’Auria A., Coratti E., Maddaloni L., Bugani G., Gentile M., Pierangeli A., d’Ettorre G. (2024). An Update on the Entomology, Virology, Pathogenesis, and Epidemiology Status of West Nile and Dengue Viruses in Europe (2018–2023). Trop. Med. Infect. Dis..

[3] Kularatne S.A., Dalugama C. (2022). Dengue infection: Global importance, immunopathology and management. Clin. Med..

[4] Bellini R., Michaelakis A., Petrić D., Schaffner F., Alten B., Angelini P., Aranda C., Becker N., Carrieri M., Di Luca M. (2020). Practical management plan for invasive mosquito species in Europe: I. Asian tiger mosquito (*Aedes albopictus*). Travel Med. Infect. Dis..

[5] Klobucar A., Merdić E., Benić N., Baklaić Z., Krcmar S. (2006). First record of *Aedes albopictus* in Croatia. J. Am. Mosq. Control Assoc..

[6] Benic N., Merdic E., Zitko T., Landeka N., Krajcar D., Klobucar A., Korunic J. (2008). Research of distribution of mosquitoes *Aedes albopictus* along Croatian coast. Proceedings of the Seminar Disinfection, Disinfestation, Deratization and Protection of Stored Agricultural Products, Šibenik, Croatia, 2–4 April 2008.

[7] Zitko T., Merdic E. (2014). Seasonal and spatial oviposition activity of *Aedes albopictus* (Diptera: Culicidae) in Adriatic Croatia. J. Med. Entomol..

[8] Klobucar A., Benic N., Krajcar D., Kosanovic-Licina M.L., Tesic V., Merdic E., Vrucina I., Savic V., Barbic L., Stevanovic V. (2016). An overview of mosquitoes and emerging arboviral infections in the Zagreb area, Croatia. J. Infect. Dev. Ctries..

[9] Vilibic-Cavlek T., Janev-Holcer N., Bogdanic M., Ferenc T., Vujica Ferenc M., Krcmar S., Savic V., Stevanovic V., Ilic M., Barbic L. (2023). Current Status of Vector-Borne Diseases in Croatia: Challenges and Future Prospects. Life.

[10] Klobučar A., Kavran M., Petrinić S., Curman Posavec M. (2024). Temporal Activity and Distribution of the Invasive Mosquitoes *Aedes albopictus* and *Aedes japonicus* in the Zagreb Area, Croatia. Trop. Med. Infect. Dis..

[11] UpToDate. Dengue Virus Infection: Clinical Manifestations and Diagnosis. https://www.uptodate.com/contents/dengue-virus-infection-clinical-manifestations-and-diagnosis.

[12] Khan M.B., Yang Z.S., Lin C.Y., Hsu M.C., Urbina A.N., Assavalapsakul W., Wang W.H., Chen Y.H., Wang S.F. (2023). Dengue overview: An updated systemic review. J. Infect. Public Health.

[13] European Center for Disease Control and Prevention (ECDC): Annual Epidemiological Reports (AERs). https://www.ecdc.europa.eu/en/publications-data/monitoring/all-annual-epidemiological-reports.

[14] European Center for Disease Control and Prevention (ECDC) Local Transmission of Dengue Virus in Mainland EU/EEA, 2010–Present. https://www.ecdc.europa.eu/en/all-topics-z/dengue/surveillance-and-disease-data/autochthonous-transmission-dengue-virus-eueea.

[15] European Center for Disease Control and Prevention (ECDC) 12-Month Dengue Virus Disease Case Notification Rate per 100 000 Population, November 2023 to October 2024. https://www.ecdc.europa.eu/en/publications-data/12-month-dengue-virus-disease-case-notification-rate-100-000-population-1.

[16] Gjenero-Margan I., Aleraj B., Krajcar D., Lesnikar V., Klobučar A., Pem-Novosel I., Kurečić-Filipović S., Komparak S., Martić R., Duričić S. (2011). Autochthonous dengue fever in Croatia, August-September 2010. Euro Surveill..

[17] Pem-Novosel I., Vilibic-Cavlek T., Gjenero-Margan I., Kaic B., Babic-Erceg A., Merdic E., Medic A., Ljubic M., Pahor D., Erceg M. (2015). Dengue virus infection in Croatia: Seroprevalence and entomological study. New Microbiol..

[18] Becker N., Petric D., Zgomba M., Boase C., Madon M., Dahl C., Kaiser A. (2020). Mosquitoes: Identification, Ecology and Control.

[19] Leparc-Goffart I., Baragatti M., Temmam S., Tuiskunen A., Moureau G., Charrel R., de Lamballerie X. (2009). Development and validation of real-time one-step reverse transcription-PCR for the detection and typing of dengue viruses. J. Clin. Virol..

[20] European Center for Disease Control and Prevention (ECDC) EU Case Definitions. https://www.ecdc.europa.eu/en/all-topics/eu-case-definitions.

[21] European Center for Disease Control and Prevention (ECDC) Dengue Worldwide Overview. https://www.ecdc.europa.eu/en/dengue-monthly.

[22] Sacco C., Liverani A., Venturi G., Gavaudan S., Riccardo F., Salvoni G., Fortuna C., Marinelli K., Marsili G., Pesaresi A. (2024). Marche dengue outbreak group. Autochthonous dengue outbreak in Marche Region, Central Italy, August to October 2024. Euro Surveill..

[23] Croatian Meteorological and Hydrological Service. https://www.meteo.hr/index_en.php.

[24] Liu Z., Zhang Q., Li L., He J., Guo J., Wang Z., Huang Y., Xi Z., Yuan F., Li Y. (2023). The effect of temperature on dengue virus transmission by *Aedes* mosquitoes. Front. Cell. Infect. Microbiol..

[25] World Health Organization Dengue–Global Situation. https://www.who.int/emergencies/disease-outbreak-news/item/2024-DON518.

[26] Ciota A.T., Chin P.A., Ehrbar D.J., Micieli M.V., Fonseca D.M., Kramer L.D. (2018). Differential effects of temperature and mosquito genetics determine transmissibility of arboviruses by *Aedes aegypti* in Argentina. Am. J. Trop. Med. Hyg..

[27] Trejo I., Barnard M., Spencer J.A., Keithley J., Martinez K.M., Crooker I., Hengartnet N., Romero-Severson E.O., Manore C. (2023). Changing temperature profiles and the risk of dengue outbreaks. PLoS Clim..

[28] Petrić M., Ducheyne E., Gossner C.M., Marsboom C., Nicolas G., Venail R., Hendrickx G., Schaffner F. (2021). Seasonality and timing of peak abundance of *Aedes albopictus* in Europe: Implications to public and animal health. Geospat. Health.

[29] European Climate and Health Observatory Dengue. https://climate-adapt.eea.europa.eu/en/observatory/evidence/health-effects/vector-borne-diseases/dengue-factsheet.

[30] Brem J., Elankeswaran B., Erne D., Hedrich N., Lovey T., Marzetta V., Salvado L.T., Züger C., Schlagenhauf P. (2023). Dengue “homegrown” in Europe (2022 to 2023). New Microbes New Infect..

[31] Procopio A.C., Colletta S., Laratta E., Mellace M., Tilocca B., Ceniti C., Urbani A., Roncada P. (2024). Integrated One Health strategies in Dengue. One Health.

[32] Lee M.F., Long C.M., Poh C.L. (2024). Current status of the development of dengue vaccines. Vaccine X.

