# Supplementary material for: Epidemiological and Entomological Study After the Possible Re-Emergence of Dengue Fever in Croatia, 2024
Source: Microorganisms. 2025 Mar 2;13(3):565. doi: 10.3390/microorganisms13030565 (PMC11944314; doi:10.3390/microorganisms13030565)
Supplement: Supplementary file 1 [file microorganisms-13-00565-s001.zip › microorganisms-3478439-supplementary.pdf]

**Table S1.** Results of flavivirus serology testing for cross-reactivity

| Case | TBEV | | WNV | | JEV | | YFV | | USUV |
|---|---|---|---|---|---|---|---|---|---|
| | IgM | IgG | IgM | IgG | IgM | IgG | IgM | IgG | IgG |
| 1 | Negative | Negative | Negative | Negative | Negative | Negative | Negative | Negative | Negative |
| 2 | Negative | Negative | Negative | Negative | Negative | Negative | Negative | Negative | Negative |
| 3 | Negative | Negative | Negative | Negative | Negative | Negative | Negative | Negative | Negative |
| 4 | Negative | Negative | Negative | Negative | Negative | Negative | Negative | Negative | Negative |
| 5 | Negative | Negative | Negative | Negative | Negative | Negative | Negative | Negative | Negative |
| 6 | Negative | Negative | Negative | Negative | Negative | Negative | Negative | Negative | Negative |
| 7 | Negative | Negative | Negative | Negative | Negative | Negative | Negative | Negative | Negative |
| 8 | Negative | Negative | Negative | Negative | Negative | Negative | Negative | Negative | Negative |
| 9* | Negative | Negative | Negative | Negative | Negative | Negative | Negative | 100 | Negative |

TBEV=tick-borne encephalitis virus; WNV=West Nile virus; JEV=Japanese encephalitis virus; YFV=yellow fever virus; USUV=Usutu virus; *History of YFV vaccination
